# Traumatic Bone Cyst of the Anterior Mandibular Region: A Case Report

**DOI:** 10.7759/cureus.31315

**Published:** 2022-11-10

**Authors:** Ashwini Dhopte, Prateek Tandon, Mahesh Shenoy, Rachita Mustilwar, Nishath Sayed Abdul, Hiroj Bagde, Ramanpal Singh

**Affiliations:** 1 Department of Oral Medicine and Radiology, Rama Dental College and Research Centre, Kanpur, IND; 2 Department of Prosthodontics, Rama Dental College and Research Centre, Kanpur, IND; 3 Department of Oral and Maxillofacial Surgery (OMFS) and Diagnostic Sciences, College of Dentistry, Riyadh Elm University, Riyadh, SAU; 4 Department of Periodontology, Rural Dental College, Pravara Institute of Medical Sciences (PIMS), Loni, IND; 5 Department of Periodontology, Rama Dental College and Research Centre, Kanpur, IND; 6 Department of Oral Medicine and Radiology, New Horizon Dental College and Research Institute, Bilaspur, IND

**Keywords:** panoramic radiograph, curettage, traumatic bone cyst, mandible, idiopathic bone cyst

## Abstract

A traumatic bone cyst (TBC) is an unusual non-neoplastic pseudocystic cavity in the bone that is often asymptomatic and slow-growing. It is unexpectedly detected by regular radiography imaging. These lesions are more common in the mandible than they are in the maxilla, and they are often seen in patients older than 40 years of age. A radiolucent unilocular lesion with scalloped margins is the most common radiographic appearance. If the hollow is found to contain blood or straw-colored fluid, surgical exploration is the only way to make a conclusive diagnosis of this uncommon condition. We present a case of an asymptomatic, incidentally diagnosed (on radiograph) traumatic bone cyst in a young patient involving the mandibular anterior region with periapical radiolucency. The case was diagnosed by radiographs and histopathological evaluation.

## Introduction

Primary non-odontogenic bone lesions in the jaws of adults and children are rare. A benign pseudocystic cavity in the jaw is known as a traumatic bone cyst (TBC) [1]. TBC was initially recognized as a distinct disease entity [2]. An epithelium-free lesion is solitary with no epithelial lining, enclosed by bone walls, and either missing or harboring fluids. TBC is classified by the World Health Organization (WHO) as a non-neoplastic osseous lesion since it lacks an epithelial coating, which distinguishes it from real cysts in children under the age of three [3]. A category of lesions is associated with the bone, including aneurysmal bone cysts and osseous dysplastic lesions such as central giant-cell granulomas and cherubism and TBCs. Extravasation growth, moderate bone pit, fundamental bone blister, and unicameral bony sore are a few of the other names for TBC that have been recorded in the writings of the past. A possible cause of intraosseous hematoma development is trauma to the jaw [4-6]. Unorganized and unrepaired hemorrhages may liquefy, leaving a hollow or fluid-filled bone cavity. In addition, there are other possibilities, such as aberrant development or metabolism of the jawbone area, tumor degeneration, and calcium metabolism disturbance [7].

Most individuals are diagnosed with this condition in their 20s or 30s when symptoms first appear. However, while some studies found no gender predilection, others have indicated a male predominance [8,9]. Most typically, it may be seen in the posterior mandible of the jaw, although it can also be present in other parts of the jaw (such as the anterior maxillary bone and the ramus). Most of the time, the pseudocyst is discovered during a routine X-ray screening and does not cause any symptoms. A radiographically traumatic bone cyst is radiolucent and has well-defined uneven or scalloped margins [10,11]. Cortical plate enlargement is rare when the disease is contained inside the medullary bone. The resorption of a tooth is very unusual [12,13]. A histopathologist may examine the bone and occasional sinewy tissue that has been curetted from the empty wall of a vacant bone pit during surgery since there is no epithelial layer to protect it. It is possible to see blood or a straw-colored fluid in the cavity. The curettage of the bone walls is a simple procedure that usually takes 6-12 months to recover from. Thus, postoperative radiological and clinical evaluation is recommended [14], to exclude the possibility of further disease. The curettage of the bone wall causes bleeding, which in turn leads to the production of a new bone. If the lesion is a traumatic bone cyst, the histological investigation of any available tissue will confirm its diagnosis, as will the lack of epithelial lining detected during surgical exploration. Histopathological analysis, on the other hand, may not always be achievable due to a lack of or a sparse quantity of examination material. As a result, surgery is the most common way to make a definitive diagnosis of a traumatic bone cyst. Three to six months after surgery, the first signs of healing may be seen; full recovery is predicted to take two years. Because of recurrence (between 2% and 26%), radiographic and clinical follow-up is strongly advised. This case report shows a young adult with a traumatic bone cyst in the front of the mandible that was found by accident and has no symptoms.

## Case presentation

A young adult in his early 20s presented to the department of oral medicine and radiology complaining of misaligned teeth. Informed consent was taken, and ethical clearances were taken from the ethical committee of New Horizon Dental College and Research Institute with reference number NHDCRI/2017-245. The patient's medical history and dental history do not have relevant findings. Swelling or lymphadenopathy in the neck or submandibular region was not seen during the extraoral examination. On clinical examination, the patient was completely asymptomatic; only some spacing between the maxillary anterior teeth was observed.

**Figure 1:**
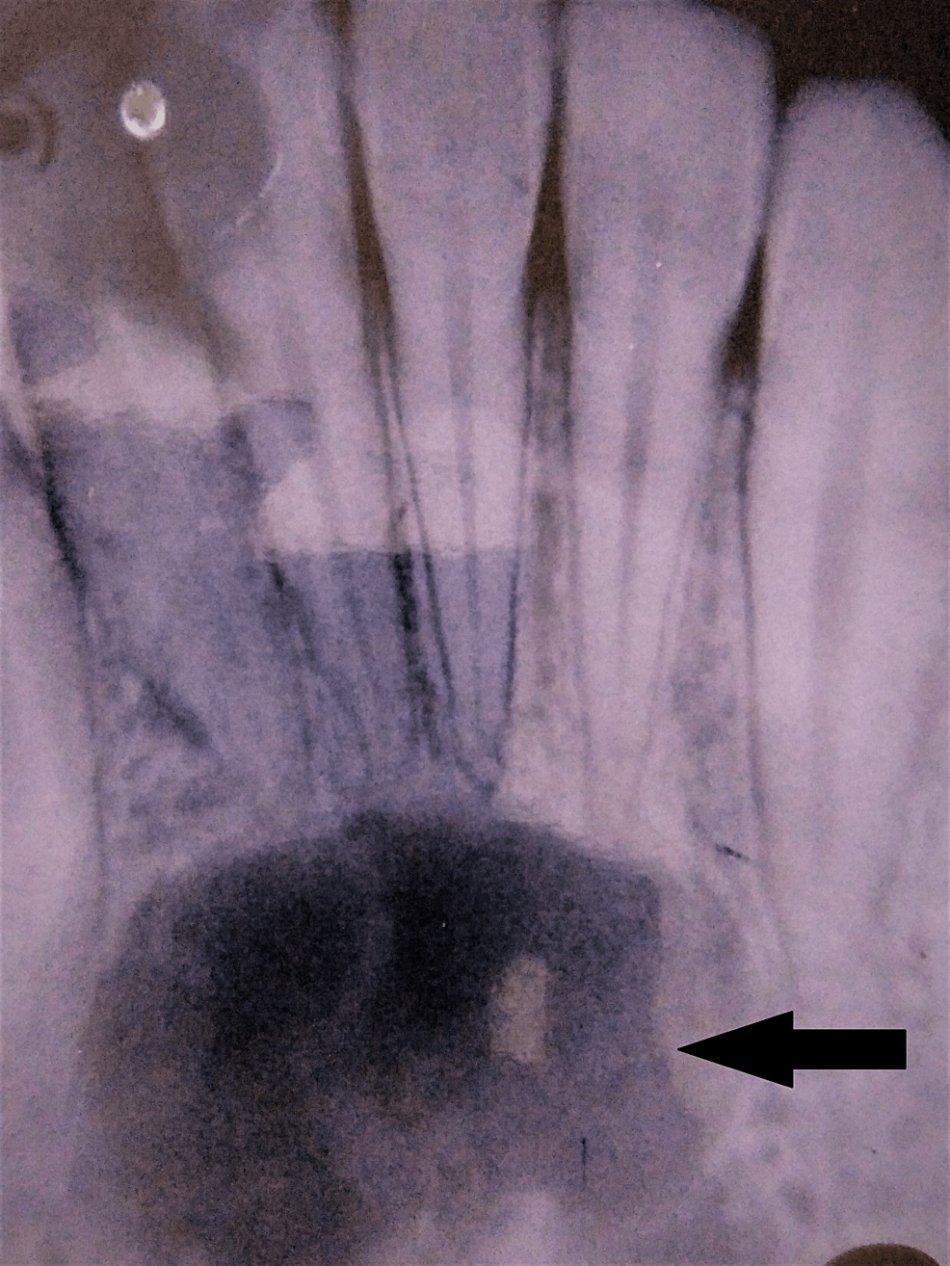
Cyst shown in IOPA radiograph IOPA: intraoral periapical

All teeth showed a positive vitality test. Considering the history and clinical findings, a provisional diagnosis of Angles class I malocclusion with maxillary anterior spacing was made. The patient was given the recommendation to have a panoramic radiography examination in preparation for orthodontic treatment; nevertheless, the unintentional discovery of the radiolucency, which was connected to the mandibular anterior area, was made. The patient reported that they had no prior knowledge of the lesion and that they had not seen any symptoms. Extraoral checks revealed no edema this time around either. Preliminary radiographic evaluation of the periapical radiolucency revealed a midline unilocular radiolucency at the apex of 31-33 and 41-43. The borders are well defined, and the internal structure is radiolucent. There was no evidence of root resorption or tooth displacement. The mandible's lower border was noticeably thinner when it was studied on the computed tomography (CT) scan. Traumatic bone cysts and periapical cemento-osseous dysplasia were among the possible differential diagnoses based on the imaging results. Figure 1 shows an intraoral periapical radiograph with a cyst present.

**Figure 2:**
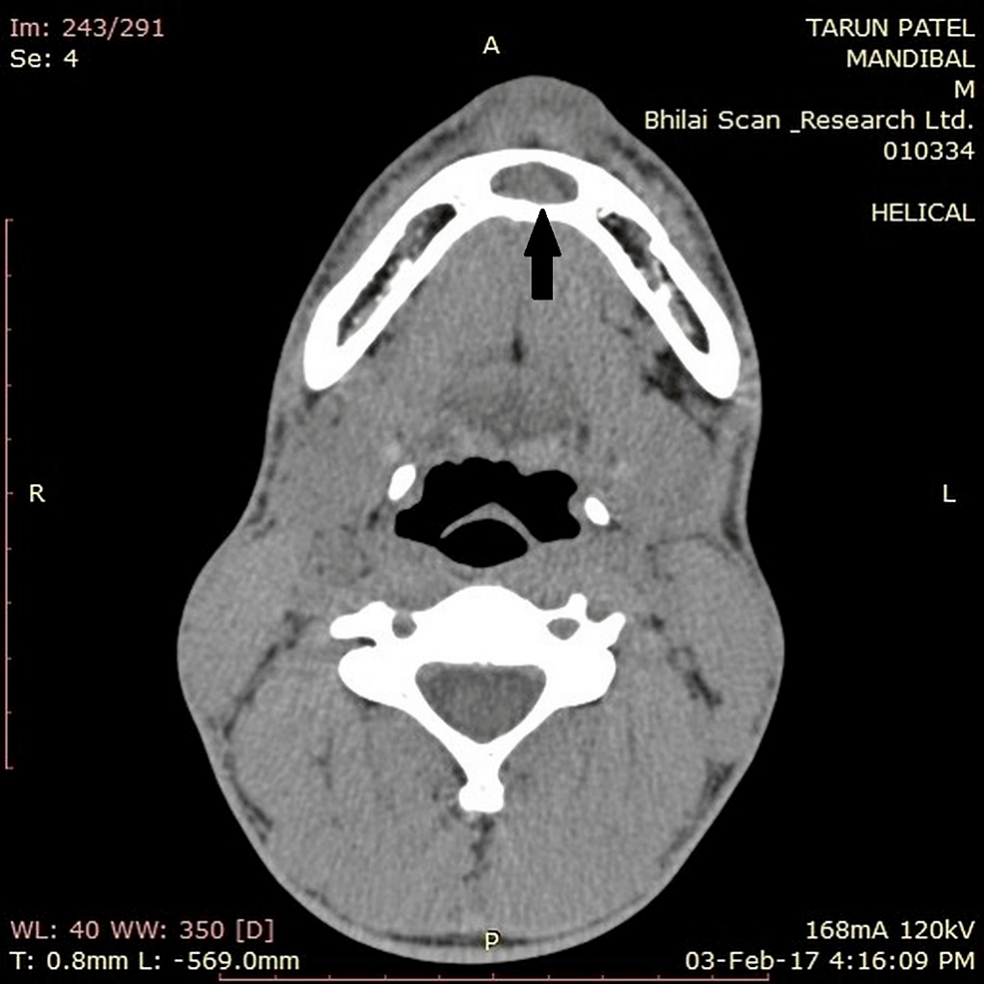
CT scan showing the cyst in the anterior region CT: computed tomography

The mandibular anterior region has a cyst-like hypodense area on a computed tomography scan (axial view) (Figure 2).

An odontogenic cyst or tumor was ruled out as a possibility in the differential diagnosis (probably mural or unicystic ameloblastoma). The oral and maxillofacial surgery department was called in to evaluate and treat the patient. A local anesthetic was used to conduct a bone biopsy. Three capsules of 2% lidocaine with 1:100,000 epinephrine were used to provide a local anesthetic to the patient. Using a 1.5 cm-long incision, the buccal gingiva of teeth number 33 to number 43 was removed. Dissection was performed in order to locate the lesion's cortex, and a bone window was constructed using a saber. Following a meticulous curettage, regeneration was opted by the use of bone grafts and collagen membranes. The incision site was closed with 3-0 silk suture once hemostasis has been established. The patient was released from the hospital after being assessed and found to be in good health. This diagnosis was well supported by the operational results; therefore, no additional therapy was necessary other than curettage. Inflammatory cells and fibroblasts were found in the bone's connective tissue during the histopathological analysis of the bone. Bone chips with osteocyte-filled lacunae were found. On microscopic analysis, several bone voids were found to be empty. A traumatic bone cyst diagnosis was made based on the research. There was no recurrence of the lesion while the patient was being monitored for a period of two years.

## Discussion

Traumatic bone cysts are quite uncommon. The pathogenesis of TBC is thought to include a number of different causes. It is generally agreed that trauma causes intraosseous hematomas to occur, and this is the most commonly acknowledged process. Following trauma, the blood clot dissolves, and the neighboring bone is degraded due to enzyme activity. According to Thomas' theory, osteoclastic resorption may be caused by trauma causing subperiosteal hemorrhage. It has also been suggested that bone tumor degeneration, changed calcium metabolism, and local bone growth modification are contributing factors in the development of TBC. Other possible causes include low-grade chronic infection and enhanced osteolysis, intramedullary hemorrhage, and ischemic necrosis. Our patient's TBC was idiopathic, with no known etiological causes to blame. Second-decade-of-life TBC is the most common form of the disease in young people. The overwhelming majority of TBCs in the oral cavity is found in this space between the canine and third molar. It is the second most common place [15] for a fracture [16] in the mandible. The focus of this case study was a young adult male patient, 18 years old, with a TBC in the front portion of the mandible.

These lesions are often found by accident during regular radiological examinations since they are usually not associated with any symptoms. Approximately 10%-30% of the population suffers from pain. Some of the less frequent symptoms include tooth sensitivity, paresthesia, fistulas, delayed permanent tooth eruption, inferior alveolar canal displacement, and pathologic fracture of the jaw [17]. A common occurrence is an increase in the width of the cortical plate of the mandible's jawbone. In this case, there were no symptoms and no expansion of the bone. Most of the teeth in the vicinity of the lesion seem to be healthy, with no root movement, displacement, or resorption [7,8]. The use of panoramic radiography and computed tomography (CT) scans is essential for the early detection of TBC as there was the unavailability of cone beam computed tomography in the department. The traumatic bone cyst usually shows up on radiographs as a single, radiolucent zone with an irregular but well-defined (or partly well-defined) structure, with or without a sclerotic layer encircling its perimeter. A "scalloping effect" is created when a traumatic bone cyst spreads between the tooth's roots. The scalloped edge may also be seen in those who are missing some or all of their teeth. When TBC initially begins, multilocular lesions without internal septa are prevalent. The multilocular look is caused by a sloping of the endosteal surface of the buccal or lingual cortical plate [8]. Occasionally, the cortical plate expands or is eroded.

A 15-gauge needle is advised for aspiration prior to the surgical investigation of the lesion since it is possible that the lesion contains blood vessels. An empty bone cavity without epithelial lining is the only way to be certain of the diagnosis. Sometimes, a clear, straw-colored liquid of blood might be found in the cavernous space. In the current instance, the bone cavity was found to be gushing blood. In many cases, a precise histological diagnosis is impossible due to a lack of available histological specimens [13]. Most of the results showed fibrous connective tissue and normal bone. Epithelial linings are nonexistent. Vascularity, fibrin, and erythrocytes, as well as large cells near the bone surface, may be present in the lesion. Odontogenic tumors, including ameloblastomas, should be examined in the differential diagnosis of TBC. Periapical cemento-osseous dysplasia should also be included. However, lesions exhibit indications of internal bone septa radiographically, creating a multilocular appearance in the mandibular anterior area as shown in our instance.

Periapical cemento-osseous dysplasia occurs in a wide age range, is mostly seen in young adults, shows minimal mediolateral expansion, and tends to cause resorption and displacement of the teeth. In contrast to our case, ameloblastoma is commonly seen in people 20-40 years of age in the molar ramus region of the mandible. Clinically, it shows more bone expansion than TBC and, in later stages, can perforate the bone. It has a tendency to resorb and displace the teeth, although resorption is more common. Ameloblastoma is usually multilocular with curved, coarse internal septa. A histological appearance of cancellous bone with a low liquid content that is either unlined or only very rarely lined by a thin connective tissue layer is the norm. Bone cysts caused by trauma lack an epithelial layer. Only 9.52% of the samples histologically examined had an epithelial component, indicating the presence of vascular connective tissue. This shows that one of the most distinguishing aspects of these lesions is the lack of epithelial tissue. In our instance, bony sample was retrieved for histological investigation during surgery. When the lesion is identified early, blood or serosanguineous fluid is generally present. The electrolyte and protein concentrations in the fluid taken from the cyst cavity are often comparable to those in serum. Extravasated blood may be the source of the fluid, according to some experts. The quantity of fluid in the lesion decreases with time until finally, the lesion is completely empty. The bony cavity in this instance was devoid of any fluid.

TBCs are often treated by surgical investigation followed by the removal of the bone walls using a curettage tool. Surgery is a diagnostic tool and a therapy method at the same time since it induces bleeding in the cavity. A clot is formed when blood seeps into a hollow and hardens into the bone. It is possible that a spontaneous resolution will occur in certain circumstances [18-21]. Diagnosing a traumatic bone cyst before surgery is really challenging in most cases. The curettage conducted during the treatment stimulates bleeding and additional osseous regeneration, so surgical investigation not only verifies the diagnosis but also cures it. While the curettage of the bone pit is a viable alternative, it has been shown that gel foam and platelet-rich plasma union, as well as intralesional infusions of blood, permeable hydroxyapatite, and bone pieces, are also viable options. This grafting method has been shown to have positive outcomes, while other research has questioned its effectiveness. Some patients had the lesion disappear on its own. However, simple monitoring is not suggested since certain TBCs may develop rapidly and aggressively. Cases like this one show how TBC may be diagnosed using radiographic and histopathological features, as well as how it is asymptomatic in this particular instance. There have been no more recurrences after the patient was medically operated on. As far as TBCs are concerned, there is a general consensus that their cause and pathophysiology remain a mystery. Questions about trauma's role in the formation of TBCs still need to be answered before any conclusions can be drawn about the disease's pathophysiology. Data for research on these issues can only be gathered by accurate, thorough, and comprehensive reporting of incidents.

## Conclusions

TBC is a benign non-epithelial lined cavity in the jawbones with an unknown etiology. Trauma is regarded as a major causative factor, but in our case, the origin of the lesion was idiopathic. Upon examination of this patient's bones, the pathologist determined that the patient had a traumatized bony lesion, which was confirmed by histological findings of a straw-colored fluid and bright blood flowing from the cavity. Clinical, radiographic, and histological examinations should be performed as soon as possible for a young adult with or without a history of trauma who has a bony enlargement of the mandible. The very low incidence of non-odontogenic primary bone lesions in the jaw requires doctors to investigate all options when creating a differential diagnosis. The clinical features and therapy of traumatic bony lesions are also critical in reducing patient morbidity. Surgery and curettage are needed for correct treatment and diagnosis and to rule out malignant lesions, which can grow and destroy the bone if they are not taken care of.
